# Factors attributed to violent behaviour by primary caregivers toward their relative with schizophrenia

**DOI:** 10.1192/j.eurpsy.2023.2269

**Published:** 2023-07-19

**Authors:** M. Stambouli, F. Fekih Romdhane, F. Ghrissi, W. Cherif, M. Cheour

**Affiliations:** Psychiatry department E, Razi hospital, Manouba, Tunisia

## Abstract

**Introduction:**

There is a modest but consistent association between violent behavior and schizophrenia. Persons with schizophrenia are at a modestly increased risk of committing violence ,with approximately half of victims being relatives

**Objectives:**

Our study examined the factors attributed to violent behaviour within the relationship patient-caregiver in schizophrenia according to caregivers.

**Methods:**

This is a cross-sectional study among caregivers of patients with schizophrenia during the period from June to August 2022.Patients who attended our department of psychiatry at the Razi.

The questionnaire was divided into three sections. The first section contained items regarding patient- and caregiver-related information.

In the second section, caregivers were asked questions about their experience of violence perpetration and victimization involving their relative with schizophrenia in the past 12 months.

Beyond frequency, caregivers were also asked to specify, the causes of the violence perpetrated and suffered

The third section contained two measures, i.e. the Depression Anxiety and Stress Scales (DASS-21) and the abridged version of the Zarit Burden Interview (ZBI), assessing psychological distress and caregiving burden, respectively. The protocol of the study was approved by the ethics committee of the Razi Psychiatric Hospital.

**Results:**

The majority of caregivers were females (63.6%), and consisted of patients’ parents (50.9%).

The most endorsed causes of violence victimization were symptoms of illness (57.3%), followed by refusal to adhere to treatment (49.1%), drug reaction (23.6%), and negative events; while the most reported causes of violence perpetration were refusal to adhere to treatment (42.7%), Symptoms of illness (37.3%), and limitation of patients’ activities and/or liberty (32.7%).

Bivariate analysis showed that lower patients’ economic status (p=.042), tobacco (p=.015) and alcohol use (p=.014) as well as taking Trihexyphenidyl (p=.001) were significantly and positively associated with violence perpetration by caregivers against their relatives with schizophrenia.

Multivariable analysis (Logistic regression) revealed that caregivers’ levels of burden remained significantly associated with violence victimization occurrence (p=.026; OR=1.48), while only having other person in charge of caring represented a significant factor associated with perpetration of any form of violence against patients (p=.007; OR=.17).

**Conclusions:**

It is important for medical staffs to provide caregivers with professional knowledge about patients’ real motivation for violence in order to improve their skills of problem-solving.

**Disclosure of Interest:**

None Declared

